# Medication errors and drug knowledge gaps among critical-care nurses: a mixed multi-method study

**DOI:** 10.1186/s12913-019-4481-7

**Published:** 2019-09-06

**Authors:** Juan Escrivá Gracia, Ricardo Brage Serrano, Julio Fernández Garrido

**Affiliations:** Department of nursing, University of Valencia, 46001 Jaume Roig St, Valencia, Spain

**Keywords:** Medication errors, Critical care, Error cause, Gaps in drug knowledge

## Abstract

**Background:**

Medication errors are a serious and complex problem in clinical practice, especially in intensive care units whose patients can suffer potentially very serious consequences because of the critical nature of their diseases and the pharmacotherapy programs implemented in these patients. The origins of these errors discussed in the literature are wide-ranging, although far-reaching variables are of particular special interest to those involved in training nurses. The main objective of this research was to study if the level of knowledge that critical-care nurses have about the use and administration of medications is related to the most common medication errors.

**Methods:**

This was a mixed (multi-method) study with three phases that combined quantitative and qualitative techniques. In phase 1 patient medical records were reviewed; phase 2 consisted of an interview with a focus group; and an ad hoc questionnaire was carried out in phase 3.

**Results:**

The global medication error index was 1.93%. The main risk areas were errors in the interval of administration of antibiotics (8.15% error rate); high-risk medication dilution, concentration, and infusion-rate errors (2.94% error rate); and errors in the administration of medications via nasogastric tubes (11.16% error rate).

**Conclusions:**

Nurses have a low level of knowledge of the drugs they use the most and with which a greater number of medication errors are committed in the ICU.

**Electronic supplementary material:**

The online version of this article (10.1186/s12913-019-4481-7) contains supplementary material, which is available to authorized users.

## Background

Pharmacotherapy is a very important resource within the health system context. However, it is not without patient risk and its improper use can cause a wide variety of damage, both iatrogenic in nature and those derived from mistakes made as part of the complex processes comprising the drug-use system. The term ‘drug-related problems’ is now preferred because it encompasses a much broader range of adverse drug reactions and medication errors and interactions [1–4].

The magnitude of this problem was highlighted in 1999 by the Institute of Medicine, which estimated the annual mortality due to medication errors at 7000 deaths, with clinical error being the most prevalent problem [5]. Comparison of different publications is difficult because of differences in the variables used, measurement and detection methods, and study populations, as well as the lack of an internationally standardised taxonomy that clearly defines what constitutes an error, potential error, error cause, or contributing factor [6]. Despite this, various studies estimate that incidents related to medication account for 6–12% of hospital admissions and 2 in every 1000 hospital deaths, and therefore constitute a serious public health problem [7–9].

Intensive care units (ICUs) are especially vulnerable to errors and their consequences, which are potentially more serious for ICU patients. Critically ill patients admitted to the ICU accumulate an average of 1.7 medical errors every day, and many patients suffer a potentially life-threatening error during their stay. Medication errors are the most common type of error and account for 78% of serious medical errors in the ICU [10, 11]. This is because of the critical nature of the patients in these units, the broad, dynamic, and complex pharmacotherapy used to treat them, and the organisation of the service (excessive care burdens, communication problems, frequent changes of staff, etc.), all of which is aggravated by the urgent nature of the work undertaken in these units [10–12].

However, little or nothing is said about the lack of training or experience of the professionals working in ICUs [13]. Most studies focus on the drug administration stage, and claim that most errors occur during this pharmacotherapeutic administration stage [14]. Nonetheless, how errors in the initial stages of prescription and transcription can generate later repercussions is also worth studying [7, 15].

Published work has identified certain pharmacological groups as having extensive multicausality error risks; among these, antibacterial drugs are particularly important because of their widespread use and frequency of errors in their use [16, 17]. Another high-risk group are pharmaceuticals that cannot be administered via a nasogastric tube (NGT) [18, 19] as well as high-risk medications in general [6, 20–22]. However, key determinants must first be identified in order to define effective error prevention strategies [10, 23–25].

Several authors claim that human factors (e.g., errors in dose calculation, absence of double checking, low adherence to protocols, and especially, poor drug knowledge among professionals) most strongly influence the medical error rate [6, 13, 21, 26, 27]. Effective prevention strategies currently focus on detecting failures and redesigning the system to prevent such problems based on the relationships between the causes (or individual factors) and the environment. In this sense, it is clear that human errors are a consequence of the system, rather than a specific cause of error [7, 28].

### Aims if this study

This study identifies the main medication errors that occur in the ICU at a general hospital in the city of Valencia (Spain). We analyse the causes of these errors, based on the perception of expert professionals and determine if the level of knowledge that nurses have of the use and administration of medications is related to the errors most commonly committed in this context.

## Methods

### Design

To exhaustively explore the above question, we carried out a mixed method study with an embedded or nested concurrent design. The study structured into three phases that involves collecting quantitative and qualitative data at the same time, but the quantitative data of the first phase dominated and guided the investigation, nesting other qualitative (second phase) and quantitative (third phase) techniques to allow us to a more complete and profound view of the phenomenon [29, 30].

### Sample

The study was carried out at a general resuscitation and intensive critical care unit in a tertiary-level hospital serving a population of 364,255 inhabitants. Of the total 535 beds at the centre, the ICU had 13 beds, of which 4 were in isolation rooms.

In the first phase of the study (phase 1), a random sample of 87 admitted episodes was selected from the total admissions over the year. We set the confidence level at 95%, the design effect was 1 (simple random sampling), and the accuracy was 10%. The random selection of cases was carried out through the statistical program SPSS 22. For the second phase (phase 2), a discussion group was formed which comprised four professionals with extensive healthcare experience as well as a researcher and a teacher in the field of nursing. In the last phase (phase 3), the sample comprised nurses from the unit who voluntarily gave their consent to participate in the research.

### Procedure and data collection instruments

In phase 1, we carried out an analysis of medical records (prescription and transcription records) in order to understand the baseline situation at the unit being studied. This included a sociodemographic description of the sample, an analysis of the main active ingredients in the drugs most commonly administered and their respective administration routes, high-risk medications, potential errors, potential drug interactions, and the main areas of risk. Medication errors were analysed according to the methodology proposed by the Adverse Drugs Events Prevention Study [31]. In the same way, the error (type) classification, cause, and/or contributing factor(s) were defined using the taxonomy published by the National Coordinating Council for Medication Error Reporting and Prevention [32]. To determine the existence and level of severity of potential drug interactions, we used the Multi-Drug Interaction Checker® database from Medscape (2018) [33].

A qualitative phenomenological methodology was proposed for phase 2 which allowed the detailed study of determinants that influence the causes of medication errors through the perceptions or experiences of the professionals at the frontline in the ICU under study.

In phase 3 we designed a form to describe and evaluate the level of drug knowledge nurses, and the drugs most commonly used and/or misused in critical care. No validated questionnaires designed for purposes like our own had previously been published in the literature and so we developed a tool based on our results from the previous two study phases and the input and opinions of leading professionals in the field. Finally, a questionnaire was elaborated with closed questions of multiple answers, of which the participants had to choose one.

This questionnaire was organised around five well-differentiated areas with the aim of collecting data about the sociodemographic characteristics of the sample (1), access to information (2), notification of errors (3), consideration of errors (4), and levels of drug knowledge (5).

For the design of the questions of part 1 (sociodemographic characteristics) and 2 (access to information) we base ourselves on no validated questionnaires published in the bibliography [13, 34]. Experts helped us in the elaboration of the questions of part 3 (notification of error) aimed at knowing how nurses notify an error, if they know the existence of the notification procedure of errors of the center and if they use it. In part 4 (consideration of errors) we wanted to know if the nurses knew how to differentiate between error of medication and cause of error, for this we consulted the taxonomy published by the National Coordinating Council for Medication Error Reporting and Prevention [32]. In section 5 (level of drug knowledge) from the results of phase 1, experts in pharmacology elaborated questions in relation to the drugs most used and with which the nurses committed a greater number of errors. To guide the writing of the questions in this part, no validated questionnaires published in the literature were consulted [13, 34]. Finally, a total of 13 questions were included, the topics were as follows (number of questions for each shown in brackets):

- *Pharmacology:* identification type antibiotic (18), pharmacological target (27), posology (20), interactions (25), identification high risk drugs (26).
- *Drug management:* administration routes (21, 22, 23, 24), high risk medication administration (28, 30).
- *Drug dose calculation:* dilution, concentration and infusion rate of high-risk medications (19, 29).

Successive revisions and a previous pilot test (with 15 participants) were necessary to analyze the convenience of the different questions, answers, writing, design and presentation, adapting and merging some items to adjust the response time to about 15–20 min, as well as the elimination of some questions to increase the internal consistency with a Cronbach’s alpha of 0.801. The final questionnaire is presented in the Additional file 1.

### Data analysis

The research focuses on a broad phenomenon (medication errors, their determinants or causes and the level of drug knowledge), this forces us to use a multiple methods design in which quantitative data predominate (analysis of medication errors) nesting other qualitative (determinants of medication errors) and quantitative data (the level of drug Knowledge) that complement first phase. This own differentiation in the objectives that each phase of the study aims to achieve requires us to analyze and interpret the data of each phase separately.

The quantitative data relating to phases 1 and 3 were analysed using SPSS (v22) software. Because the variables did not follow a normal distribution we used nonparametric statistical tests and searched for correlations between variables using Spearman’s linear correlation coefficient analysis. To establish possible differences between quantitative and qualitative variables, the Kruskal–Wallis test was used for variables from three or more independent groups, and the Mann–Whitney U test was used for variables from two groups.

The qualitative analysis of the data from the second phase of the study was characterized by what Valles calls “omnipresence of the analysis” [35], referring to the fact that said analytical activity occurs at all moments of the investigation; It was already present in the formulation of the problem and the design of the study, in the field work with the discussion group to try to discover important determinants or direct the conversation towards aspects that were interesting for the researcher. However, what is usually associated as equivalent to data analysis was the transcription of the conversation, the coding and recoding of text segments, as well as their ordering and regrouping. In this phase, the greatest analytical deployment and synthetic replication of the interpretive activity took place, in order to finally identify four major categories from which different subcategories emerge whose relationships are represented graphically in a conceptual map.

Initially, the analysis of the content of the transcript was carried out by three experts in the investigation of medication errors in an independent way, afterwards several commons were made until reaching a consensus in which the same codes were reached regarding certain portions of text, making various adjustments and saturating codes to reduce redundancy and variability, as well as ensuring the validity, coherence and sensitivity of the data presented [36].

### Ethical and legal considerations

The study was undertaken according to the conditions of respect for individual fundamental rights and the ethical postulates affecting biomedical research on human beings, according to the Declaration of Helsinki and the Good Clinical Practices of the European Union. In addition, the research protocol was approved by the Clinical Research Ethics Committee at General Hospital of Valencia (Spain) (authorisation number JUA-FAR-2015-01) prior to commencing the study. Likewise, the authors declare the non existence of any type of conflict of interests.

## Results

### Phase 1: review and analysis of medical records

Of the total 87 episodes admitted to the ICU, 51.7% were men; the average patient age was 57.7 ± 16.13 years and the average length of stay was 5.97 ± 7.41 days. Most of the patients (63.22%) were admitted to the unit postoperatively after complex major surgery (critical surgical patients), whereas 36.78% of the cases were admitted for a non-surgical critical problem. We analysed 2634 drug-dose units used, corresponding to 152 different main active drug ingredients, of which 23.5% were considered high-risk. Each patient received an average prescription of 14.51 medications.

For the 2634 medications administered, we detected a total of 316 potential errors, corresponding to a global medication error index (GMEI) of 1.93%. The intravenous route was the most commonly used (76.92%), followed by pressurised inhalation (8.96%), and the subcutaneous (4.82%), nasogastric (4.25%), and oral (3.42%) routes. Other routes (epidural, rectal, transdermal, sublingual, and intramuscular) were used at rates accounting for less than 1% of the total.

**Fig. 1:**
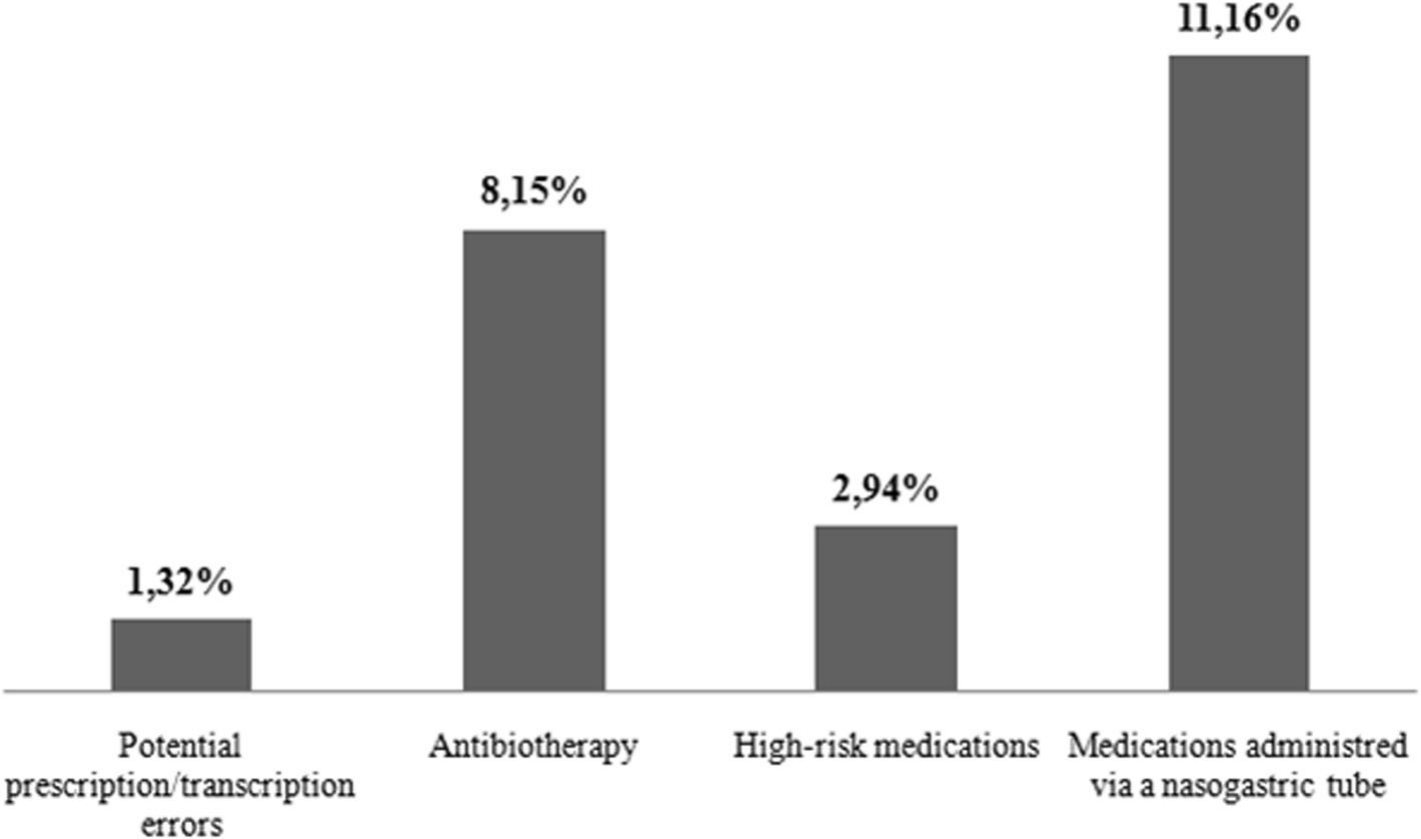
Error rate for the risk areas analysed

When reviewing the prescriptions and transcriptions for these 87 admissions, we identified an error rate of 1.32%. The most common error (accounting for 71% of the total) was the lack of a complete written prescription, while 29% of the remaining errors occurred during transcription of the prescriptions (omission of dose, incorrect dose, or erroneous dose-administration frequency or infusion rate). A more detailed analysis of the drug groups or administration techniques indicated as potential areas of risk in the literature, revealed substantially higher error rates in this study (Fig. 1).

**Fig. 2:**
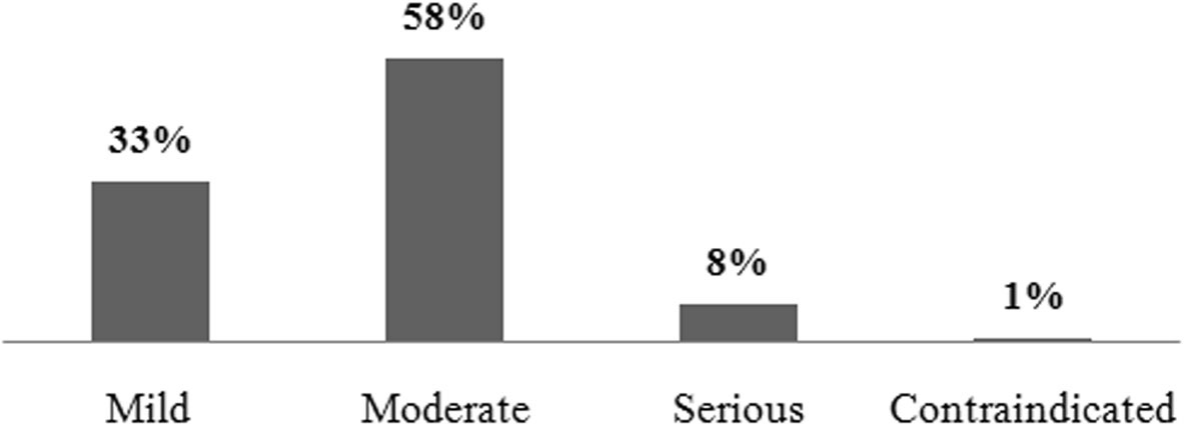
Classification of the potential drug interactions identified

Among the errors detected, deviations in the schedule of antibiotics administration—important because their efficacy is time dependent— were found in 65.9% of cases. In addition, there were significant errors in the dilution, concentration, and infusion rate used for high-risk drugs, especially those containing noradrenaline (32.9%) or potassium chloride (ClK) (47.9%) as active substances. Administration via a NGT caused errors resulting from the manipulation of oral pharmaceutical formats, with acetylsalicylic acid being involved in 32% of cases. The rate of drug interactions (Fig. 2) was also very high (f_i_ = 1811), although the clear majority of these were mild or moderate.

There were significant correlations between most of the variables analysed, and this confirmed that there is a wide range of causes behind these errors. As expected, the more drugs administered and the longer the hospital stay in the unit, the higher the probability of detecting more errors and drug interactions (*p* = 0.001 for both relationships). There was also a strong relationship between the causes of error identified in the prescription and the subsequent errors committed in the transcription of these prescriptions (*p* = 0.003); the use of abbreviations and the absence of a dose, administration route, and/or schedule specified in the prescription was the main reason for errors related to dose omission, incorrect doses or administration frequency, and/or erroneous infusion rates in the transcript.

Importantly, we also found significant differences (Mann-Whitney U test *p* < 0.05) between patients in intensive medical care versus those admitted to the critical care unit post-surgery in relation to key variables in the commission of medication errors (Table 1). Similarly, the presence of secondary diagnoses or comorbidities also affected the number of errors detected for these patients (Table 2).

**Table 1:** Significant differences between patients admitted to the unit for critical medical or post-surgical care (Mann–Whitney U test)

Variable	Average		Significance
Length of hospital stay	Medical admission	56.77	0.001
	Surgical admission	36.57	0.001
Number of drugs	Medical admission	57.72	0.001
	Surgical admission	36.02	0.001
Error causes	Medical admission	54.23	0.004
	Surgical admission	38.05	0.004
Total errors detected	Medical admission	56.52	0.001
	Surgical admission	36.72	0.001
Drug interactions	Medical admission	56.92	0.001
	Surgical admission	36.48	0.001

**Table 2:** Significant differences between the presence or absence of secondary diagnoses (Mann–Whitney U test)

Variables	Significance	Secondary diagnoses	Average
Age	0.001	Yes	50.37
		No	30.57
Number of drugs	0.007	Yes	49.03
		No	33.41
Drug interactions	0.046	Yes	47.72
		No	36.16

### Phase 2: discussion group

**Fig. 3:**
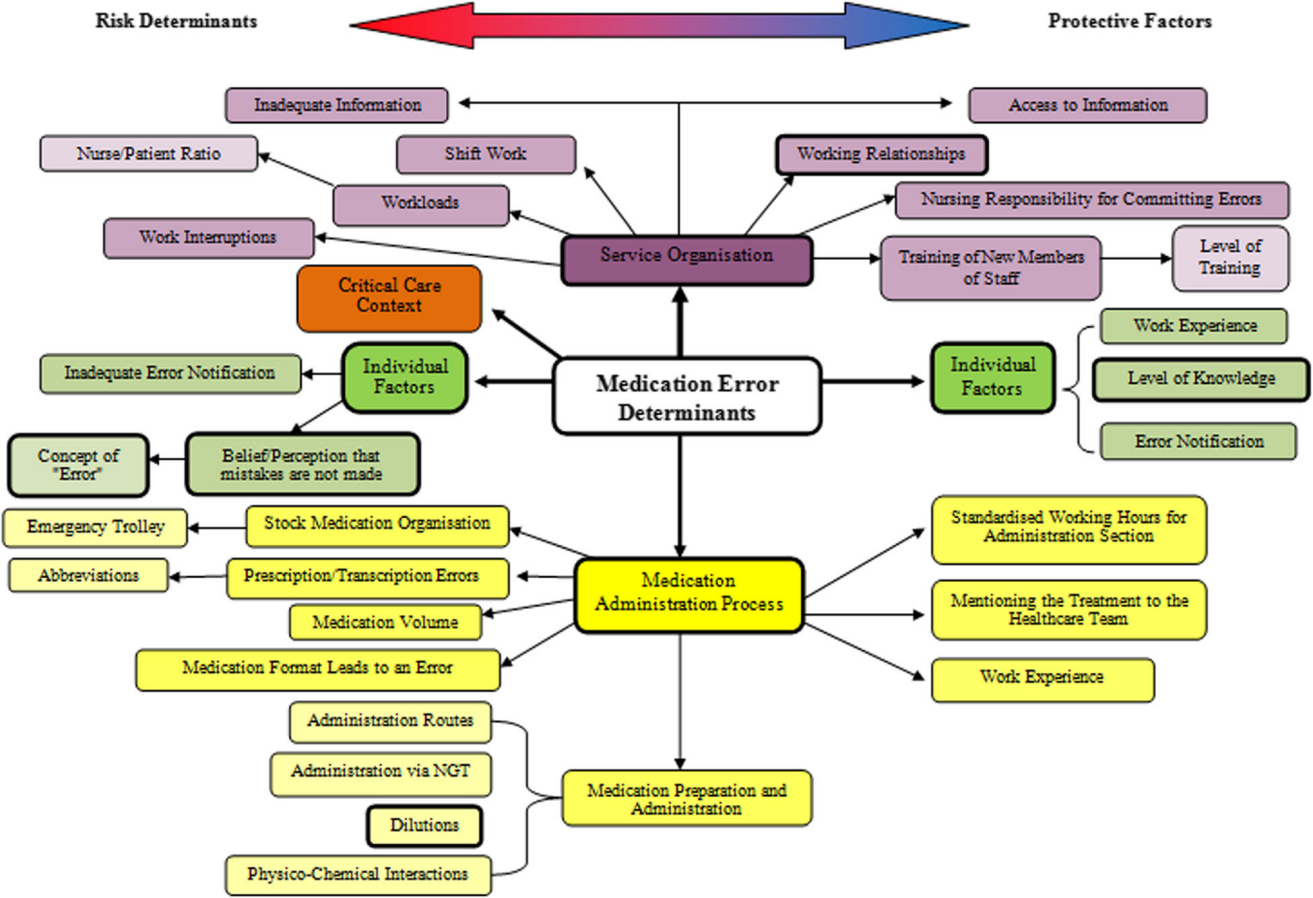
Conceptual map of the results from the focus group discussion

The information obtained from the focus group conversation allowed us to identify four major areas that lead to medication errors: (1) the critical-care context itself; (2) organisation of the ICU service; (3) personal factors; and (4) the medication administration process. These categories emerged from several subcategories which can be drawn on a broad conceptual map of interrelations. According to the professionals in the focus group these medication errors result from varying and complex multiple causes which are present in ICUs (Fig. 3). Among this wide network of subcategories, the most commonly cited were relationships in the work environment; level of professional knowledge; preparation of dilutions; and the belief or perception that mistakes are not made (also including a mistaken understanding of what constitutes an error).

### Phase 3: level of drug knowledge

The level of drug knowledge was analysed in 38 nurses (81.6% female). Of these, 75% had participated in continuing education, 15% of them specifically in pharmacology. In turn, 60% of the professionals in our sample did not know if the centre’s training program offered courses related to pharmacotherapy or error prevention. We discovered that 32.5% of the sample reported possible errors following the procedure established at the hospital, 27.5% informed the attending physician and/or pharmacy service, and the remaining 35% limited their communication to their colleagues and/or supervisor. It also became clear that professionals have an erroneous understanding of what constitutes a medication error for different variables; 85% of the participants identified the omission of a dose as an error, while only 45% identified a delay of more than 1 h in the administration of an antibiotic dose as an error.

With respect to drug knowledge, 42.5% of the nurses in our sample failed more than half the questions on the test. The average score was 47% and the highest score was 69.2%, which reveals a low level of drug knowledge among these professionals. The main drug knowledge gaps detected by this test are shown in Table 3.

**Table 3:** The main drug knowledge gaps identified among nursing professionals

Aspects of drug knowledge addressed	Percentage of errors
Preparation/Administration of insulin	92.5%
NGT administration (acetylsalicylic acid)	72.5%
Administration of low-molecular-weight heparin	60%
Dilution/Mixing (noradrenaline)	60%
Dose/Concentration/Infusion speed ​​ (potassium chloride)	50%

## Discussion

Human factors are responsible for numerous medication errors [27]. However, it has now been shown that effective prevention must focus on the system, and so the main risk factors present at different stages of the pharmacotherapeutic process must be identified and evaluated so that the system for medication use can be redesigned to be stronger and more error-proof [37].

In our study, we identified a global medication error index (GMEI) of 1.93%, slightly higher than that obtained in other large multicenter Spanish studies (1.74%). However, the lack of a common and homogeneous criterion and taxonomy that clearly defines medication errors, causes and contributing factors explains the existence of such disparate published results, with a rate that varies from 0.2 to 25.7% [3, 38–40]. In turn, this lack of criteria makes it difficult for professionals to have a clear view of the error, its magnitude and importance in clinical practice.

We identify that in the prescription stage a greater number of errors occur than in the transcription, being in this last stage much more varied the type of errors. Our results are similar to others in the literature, however, we find again disparity due to the lack of homogeneous criteria and diversity in the designs of the studies [41]. In any case, there is a significant correlation between the lack of a correct and complete written prescription and the subsequent presence of errors during transcription. Failures that occur through transcriptions (caused by the lack of a written recipe, abbreviations, etc.) are one of the most frequent causes of errors and one of the most dangerous errors because they are often not detected or not are considered subjectively as errors [23, 37].

A logical and well-studied factor that reaffirms the results obtained in our study is the correlation between the use of a greater number of drugs and a longer stay as factors that increase exposure and therefore the risk of medication errors and interactions [42–44].

The differences in the errors committed between critical patients admitted for medical versus post-surgical care are very interesting. In this sense, the specific nature of post-surgical patient critical care (the therapeutic purpose, physiopathological processes involved, their programmed admission, and continuity of care) and the relative absence of comorbidities, are intrinsic characteristics associated with shorter hospital stays and simpler pharmacotherapeutic programs with a lower risk of exposure, and therefore, of medication errors and drug interactions [42–44].

From a pharmacological point of view, together with previously published results, our findings confirm the existence of important risk areas in critical care related to the administration of medications via NGTs, although the intravenous route is the most widely used in this context [18, 19]; the dose interval of antibiotics [16, 17]; and the dilution, concentration, and infusion speed of high-risk medications [6, 21, 22, 45, 46].

It is also important to account the possible consequences derived from the numerous potential moderate-risk drug interactions that we identified in the ICU in this study [47].

In our study, the professionals identify important determinants that influence the problem (discussion group). These results coincide with those published in the literature by other authors [15]. These variables are lack of communication, poor relationships with the work environment, excessive pressure at work, interruptions in work and a misunderstanding of what constitutes an error, combined with the urgent and critical nature of the provision of professional medical attention in this context. The key variables and root causes of this problem must be continuously analysed so that specific prevention strategies can be defined and to verify their efficacy in clinical practice through successive observational studies.

The medication errors are have a wide variety of interrelated root causes, with nurses’ level of drug knowledge and/or access to information being strongly determining element [6, 13, 21, 25, 26, 48].

Although we cannot prove that increase in drug knowledge is not necessarily associated with changes in clinical practice, many published studies reach similar conclusions to us, and point out a poor of drug knowledge among nurses, especially in the context of ICUs with the drugs most used and in which accumulate a higher error rate [24, 37].

### Limitations

Our study is partially limited by the intrinsic limitations that contain the error analysis methods we have used, which are described in the literature. Another potentially important limiting factor was that our analysis sample was small and comprised exclusively nurses, to whom we passed an not validated ad hoc questionnaire. Consequently, we have not been able to make solid inferences to the general population. However, in the absence of validated questionnaires, this instrument served to provide an approximate description of the level of drug knowledge, and to reduce the possible bias, we passed our questionnaire to all the nurses of the studied ICU.

## Conclusions

A considerable number of medication errors occur in ICUs, especially with critical medical patients. The administration of medications via NGTs, the dose interval of antibiotics and the dilution, concentration, and infusion speed of high-risk medications constitute important areas of risk of medication errors in ICU. Nurses identify four major areas that lead to medication errors: the critical-care context itself; organisation of the ICU service; personal factors; and the medication administration process. In addition, the nurses have a low level of knowledge of the drugs they use the most and with which a greater number of medication errors are committed in the ICU.

## Additional file

Additional file 1: Multiple-Choice Questions. (PDF 185 kb)

## Data Availability

The datasets supporting the conclusions of this article are available in the OSF repository: https://osf.io/te7sa/.

## Abbreviations

ATC: Anatomical, Therapeutic, Chemical
ClK: Potassium Chloride
GMEI: Global Medication Error Index
ICU: Intensive Care Unit
NGT: Nasogastric Tube
