# Supplementary material for: Medication errors and drug knowledge gaps among critical-care nurses: a mixed multi-method study
Source: BMC Health Serv Res. 2019 Sep 6;19:640. doi: 10.1186/s12913-019-4481-7 (PMC6729050; doi:10.1186/s12913-019-4481-7)
Supplement: Supplementary file 1 — Multiple-Choice Questions. (PDF 185 kb) [file 12913_2019_4481_MOESM1_ESM.pdf]

1-Soci-demographic characteristics, training and experience

1-Sex

Male
Female

2-Age.

Between 21 and 25 years old
Between 26 and 35 years old
Between 36 and 45 years old
More than 45 years

3-Years worked as a nurse.

Less than 1 year
Between 1 and 5 years
Between 6 and 10 years
More than 10 years

4-Years worked in a resuscitation / critical care service.

Less than 1 year
Between 1 and 5 years
Between 6 and 10 years
More than 10 years

5-Level / Degree of studies.

Diplomat
Graduate
Specialist (Indicate which) ....
Master (Indicate which) ....
Doctor (Indicate which) ....

6-Specialized training titles (own titles).

Yes
Not

If yes, indicate in which specialty.

Surgery
Intensive Care
Pediatrics
Medical Care
Others ....

7-In the last 3 years have you taken any continuing education, attendance at conferences or training events?

Yes
Not

If yes, indicate the option:

Related pharmacotherapy
Not related to pharmacotherapy

8- Within the training program of your workplace. Are courses related to pharmacotherapy, safe medication administration or prevention of medication errors offered?

Yes, but I do not realize them
Yes, I make them
I do not know
Not

2-Access to information

9-Do you know if there is a pharmacotherapeutic guide or form in your workplace?

Yes, but I do not have access or I have it in my work unit
Yes, although I do not use it
Yes, and I use it
No. I do not know

10-Do you know and have easy and fast access to the recommendations that can be made about a drug in the prescription and / or pharmaceutical validation of it?

Yes, I know them and I have easy access
Yes, but access to them is expensive
Not
I do not know

11- Do you respect and comply with the administration recommendations that may be given in the prescription and / or validation of a drug?

Yes, always.
Yes, as long as it does not interfere in the schedule, guidelines, rules or customary habits and consensus of all the professionals of the unit
Almost always
Seldom

12- What is your source of information about possible doubts about a drug?

Consult the prospectus and / or technical sheet of the same
Ask a coworker or doctor
Ask the pharmacist or pharmacy service
Consult administration protocol, form and / or pharmacotherapeutic guide

3-Reporting errors

13-Is there a stipulated procedure for the notification of medication errors in your workplace?

Yes, and I use it
Yes, I know it, but I do not use it
Yes, I know it exists, but I do not know the procedure
No. I do not know

14-Faced an error. How do you notify it?

Following the established procedure
I write it in the clinical history
I tell my colleagues and / or supervisor

I tell the pharmacist and / or doctor
I do not say anything

4-Error consideration

15- Do you consider the delay, in more than half an hour, in the administration of a dose of intravenous antibiotic therapy a medication error?
Yes
Not

16-Do you consider the omission of a dose a medication error?
Yes
Not

17-Do you consider the lack or inadequate registration (abbreviations, illegible, without specifying dose, schedule ...) of the medication administered, as a medication error?
Yes
Not

5-Drug knowledge

18-A patient allergic to penicillin enters our unit. Next, we observed that he has prescribed amoxicillin 2 G every 8 hours intravenous. Point out the correct option:
We administer amoxicillin, since this antibiotic does not belong to the family of penicillins
We adjusted the prescription to a schedule of administration of medication agreed upon in the unit (8-16-24 hours)
We do not administer amoxicillin, since the patient may have an anaphylactic reaction
We administer a test dose to assess your sensitivity to the drug.

19-A high-risk drug whose use is very common in critical care is potassium chloride. Point out the wrong option:
The maximum recommended amount is 150mEq per day
The maximum recommended infusion rate is 20mEq / h
The maximum recommended concentration is 40mEq / L
All are correct

20- What is the maximum recommended dose of acetaminophen per day?
3 gr / day
4gr / day
In chronic alcoholics 2gr / day
The second and third options are correct

21- Regarding the acetylsalicylic acid 100 mg gastroresistant tablet. If we need to crush the tablet to administer it by nasogastric tube in a patient with deterioration of swallowing. What we must take into account:
It can be crushed to be administered without any problem since it is 100 mg, however it cannot be in the pharmaceutical forms of 500 and 300 mg

It can be crushed taking into account that it accelerates its absorption and ulcer risk
If we crushed the tablet its bioavailability would be seriously altered
If it cannot be administered orally due to deterioration of swallowing, we should administer it sublingually

22- In relation to the preparation and administration of insulin. Point out the correct option:
The insulins with the fastest action profile (lispro, aspart ...) can be mixed with the NPH insulin
It is advisable to administer each type of insulin in separate syringes, avoiding mixing them
Human insulin and NPH can be mixed, only if they have the same pH
All of the above is correct

23-Many patients are prescribed low-molecular-weight or fractionated heparin for curative or preventive purposes. These are presented in syringes pre-loaded with different doses. Point out the wrong option:
Do not eliminate the air bubble
Administer in the supine position
Administer in anterolateral or posterolateral abdomen
Requires analytical control to establish a safe dosage

24-As you know, salbutamol is a medicine that is used in the treatment of severe bronchospasm and other processes associated with reversible airway obstruction. Point out the wrong option:
There are pharmaceutical forms for inhalation and intravenous administration
Salbutamol, solution for inhalation by nebulization, can be used undiluted, administering 2 ml of salbutamol (5 mg / ml) for about 3-5 minutes
The doses administered by inhalation are superior to those administered intravenously
The pharmaceutical form for inhalation administration can be used intravenously if diluted at a ratio of 1: 1000

25-Of the following interactions between active ingredients that we list. Which of them would be classified as serious and therefore it would be necessary to look for an alternative?
Omeprazole-Lorazepam
Furesomide-Amiodarone
Potassium chloride-Spironolactone
Atorvastatin-Diltiazem

26- Of the therapeutic groups listed below, which of them would not be classified as high risk drugs?
Oral anticoagulants
Antiarrhythmics
Antibiotics
Oral antidiabetics

27- Regarding furosemide: What is your action target?

Nephron
Arteries
Heart
All of the above

28-The use of 2% propofol as a general anesthetic for the sedation of patients in critical care with assisted ventilation is frequent. In relation to the administration of propofol, point out the incorrect option:

Both the infusion systems and the bottle should be replaced every 12 hours, and the infusion should not be maintained for more than 7 days
It may appear especially at the start of administration: bradycardia, hypotension, flushing, hyperventilation and cough
The maintenance of the sedation of an adult of 60 Kg in intensive care is obtained satisfactorily with a continuous perfusion in an interval of 15-18 ml / h
It should not be administered in patients with hypersensitivity to soy

29- Norepinephrine is a vasopressor frequently used in the pharmacotherapy of critical patients. Indicate which substance should not be diluted or mixed during your infusion

Sodium chloride 09,%
5% Glucose
Sodium bicarbonate
The first and third options

30- Remifentanil is an analgesic with sedative effects used in critical care units. Point out the wrong option:

The use of remifentanil implies less need for doses of other concomitant sedatives
The withdrawal of remifentanil should be gradual, at least 1 hour, including alternative analgesia before stopping the infusion
The effect of remifentanil disappears after 90 minutes of removing the infusion
Remifentanil treatment should not be used for more than 3 days
